# P-178. Cost Savings of Carba-R Gene Xpert® Testing for Targeted Treatment Compared to Empiric Ceftazidime-Avibactam/Aztreonam Combination for Carbapenem-Resistant Klebsiella pneumoniae in a Resource-Limited Setting with a High Prevalence of blaNDM

**DOI:** 10.1093/ofid/ofaf695.402

**Published:** 2026-01-11

**Authors:** Edsel Maurice Salvana, Jonnel B Poblete

**Affiliations:** Institute of Molecular Biology and Biotechnology, National Institutes of Health, University of the Philippines, Manila, National Capital Region, Philippines; Philippine General Hospital, University of the Philippines, Manila, Manila, National Capital Region, Philippines

## Abstract

**Background:**

The Philippines has seen a significant increase in carbapenem resistance (CR) in *Klebsiella pneumoniae* in recent years, with a current nationwide CR rate of nearly 17%. A recent study by our group revealed that 92% of carbapenem-resistant *Klebsiella pneumoniae* (CR-*Kp*) isolates harbor at least one carbapenemase gene, with the following distribution: *bla*_NDM_ (68.8%), *bla*_OXA-48-like_ (38.5%), and *bla*_KPC_ (28.6%). Standard treatment of CR-*Kp* with *bla*_NDM_ is with combination ceftazidime/avibactam and aztreonam, which is prohibitively expensive in our setting. Most Filipinos have no private insurance and public health insurance pays a small percentage of actual health costs, with the rest being out of pocket. Treatment of *bla*_KPC_ and *bla*_OXA-48-like_ can be done with ceftazidime/avibactam monotherapy. We determined whether the significant cost of molecular testing can be offset by targeted monotherapy as opposed to empiric combination therapy for all CR-*Kp* infections.

**Methods:**

We set antibiotic costs based on retail prices of each antibiotic in our pharmacy and set molecular testing costs based on commercial rates. We compared an empiric treatment scenario with targeted testing based on the previous carbapenemase gene distributions. We calculated cost savings for every 100 CR-*Kp* patients who underwent molecular testing compared to empiric therapy using a standard 7-day course of antibiotics.

**Results:**

**Table:** 

Scenario	Number of patients to be treated (n = 100)	Cost of 7-day course (in USD*)^#^	Cost of Carba-R test (in USD)	Cost (in USD)
Scenario 1: Empiric CZA/ATM for all patients	100CZA/ATM	396,312.47	No testing	396,312.47
Scenario 2: Directed therapy	63 NDMCZA/ATM	249,676.85	9,027.40	258,704.26
	37 non-NDM CZA only	120,749.53	5,301.81	126,051.34
Total cost of directed therapy				384,755.60
Cost savings with directed therapy per 100 patients tested				11,556.87

*1 USD = 55.83 PhP as of April 30, 2025

^#^CZA cost/individual = 3,263.50 USD, ATM cost/individual = 699.62 USD

^&^Carba-R cost/individual = 143.29 USD

**Conclusion:**

The cost of testing with Carba-R Gene Xpert® among CR-*Kp* -infected Filipino inpatients is more than offset by the added costs of empiric treatment, in addition to indirect benefits of improved antimicrobial stewardship and decreased downstream rates of resistance.

**Disclosures:**

All Authors: No reported disclosures

